# Sex-specific association between the gut microbiome and high-fat diet-induced metabolic disorders in mice

**DOI:** 10.1186/s13293-020-0281-3

**Published:** 2020-01-20

**Authors:** Chao Peng, Xinbo Xu, Yanshu Li, Xueyang Li, Xiaoyu Yang, Hongyan Chen, Yin Zhu, Nonghua Lu, Cong He

**Affiliations:** 1 Department of Gastroenterology, The First Affiliated Hospital of Nanchang University, 17 Yong Waizheng Street, Donghu District, Nanchang, 330006 Jiangxi Province China; 2 Jiangxi Supervision and Inspection Center for Medical Devices, Nanchang, 330029 Jiangxi China

**Keywords:** Sex difference, High fat diet, Metabolic disorders, Antibiotic, Gut microbiota, 16S rRNA gene sequencing

## Abstract

**Background:**

Accumulating evidence indicates that high-fat diet (HFD)-induced metabolic disorders are associated with dysbiosis of the gut microbiota. However, the sex-specific characteristics of the gut microbiota and its association with a sexually dimorphic response to a HFD remain unclear.

**Methods:**

Male and female mice were randomly assigned to receive a chow diet (CD) or HFD for 12 weeks. A group of HFD mice were pretreated with antibiotic cocktails for 4 weeks. Body weight, insulin sensitivity and the levels of serum metabolic parameters (blood glucose and insulin) were evaluated. 16S rRNA gene sequencing was performed to analyze the composition of the gut microbiota.

**Results:**

HFD-induced body weight gain (BWG) was higher in male mice than in female mice. While insulin resistance was increased in the HFD group compared to CD group in male mice, there was no difference in insulin resistance among female mice. Antibiotic-pretreatment alleviated HFD-induced insulin resistance in male mice and elevated fasting blood glucose in female mice. The composition of the gut microbiota in male mice was remarkably different from that in female mice independent of diet. A higher abundance of the genera *Parabacteroides*, *Lactobacillus*, *Bacteroides*, and *Bifidobacterium* was observed in females than inmales. HFD feeding also influenced the structure of the gut microbiota, as it decreased the abundance of short-chain fatty acids-producing bacteria including *Roseburia* and *Lachnospiraceae_NK4A136_group*. Alterations in the gut microbiota in response to antibiotics followed by HFD were different between males and females, indicating sex-dependent sensitivity to antibiotics.

**Conclusions:**

We identified that sex had a greater impact on the composition of gut microbiota than environmental factors (HFD and antibiotics). The enrichment of beneficial microbes in female mice may be associated with the resistance of female mice to HFD-induced metabolic disorders, which was weakened by antibiotic pretreatment.

## Introduction

Obesity and the subsequent metabolic disorders, which are associated with lifestyle changes characterized by excess energy intake and reduced physical activity [1], are topics that have inevitably gained much attention worldwide. A high-fat diet (HFD) is considered one of the most important environmental factors that contribute to the global obesity epidemic. Interestingly, significant metabolic and phenotypic differences in obesogenic environments exist between the sexes in both humans and animal models. Compared to males, females tend to have greater insulin sensitivity, and a higher degree of adiposity is required in females to achieve the same metabolic disturbances [2]. Another in vivo study showed that male mice on an HFD displayed higher blood glucose levels and insulin levels and elevated fat mass compared to those in females, which demonstrated that sex is a significant modifier of the impact of an HFD [3]. Moreover, postmenopausal females exhibit an elevated risk of developing metabolic disorders due to fluctuating levels of circulating androgens and estrogens, indicating the important role of sex hormones in this process [4]. However, the underlying mechanisms of sex dimorphism in metabolic dysfunction are unclear.

There is growing evidence that the gut microbiota plays a key role in regulating host metabolism and its causal role in obesity and insulin resistance has been demonstrated in mice [5]. Diet has recently been recognized as an important external factor in the homeostasis of the gut microbial profile. It was reported that HFD feeding induced widespread changes in the gut microbial community structure, with increased abundance of *Firmicutes* and *Proteobacteria* and decreased abundance of *Bacteroidetes* and *Actinobacteria*; these changes were significantly associated with metabolic parameters [6]. The critical effect of the gut microbiota in metabolic disorders has also been demonstrated in germ-free mice, which displayed resistance to HFD-induced obesity and insulin resistance [7]. In addition to environmental factors, host factors, including genetics and hormones, are associated with variation in the gut microbiome [8, 9]. Sex differences in the composition of the gut microbiome were observed in both humans and rodents [9, 10]. Nevertheless, little information is available regarding the interaction between diet and sex in regulating gut microbiome and the host metabolism.

Antibiotics, an important therapeutic intervention for infectious diseases, have been demonstrated to induce changes in the gut microbiota that subsequently affect host metabolism and physiology [11]. The impact of antibiotics on the gut microbiota is influenced by host-related factors (age, lifestyle, and baseline microbiota composition) and drug-related factors (antibiotic class, exposure time, and route of administration) [12]. Extensive studies have shown profound compositional changes in the gut microbiota after short-term antibiotic treatment, with a remarkable reduction in taxonomic diversity and richness [13, 14]. A recent study investigated the long-lasting effects of antibiotics and reported that mice that received subtherapeutic doses of antibiotics in early life exhibited a significant increase in weight [15]. However, antibiotics can also act positively on the gut microbiota by stimulating the growth of beneficial bacteria [12]. Thus, antibiotics may be a double-edged sword in clinical practice, and their eubiotic or dysbiotic effect depends on the situation.

In the present study, we fed male and female mice an HFD or standard chow diet for 12 weeks and evaluated metabolic parameters. Another group of HFD-fed mice was pretreated with antibiotic cocktails including vancomycin, neomycin, metronidazole, and ampicillin for 4 weeks to observe the role of the gut microbiota in sex-specific susceptibility to an HFD. The gut microbial composition was analyzed by 16S rRNA gene sequencing. The aim of this study was to carry out a sex-specific characterization of the gut microbiota and identify its association with HFD-induced metabolic disorders.

## Materials and methods

### Animals and diets

**Fig. 1:**
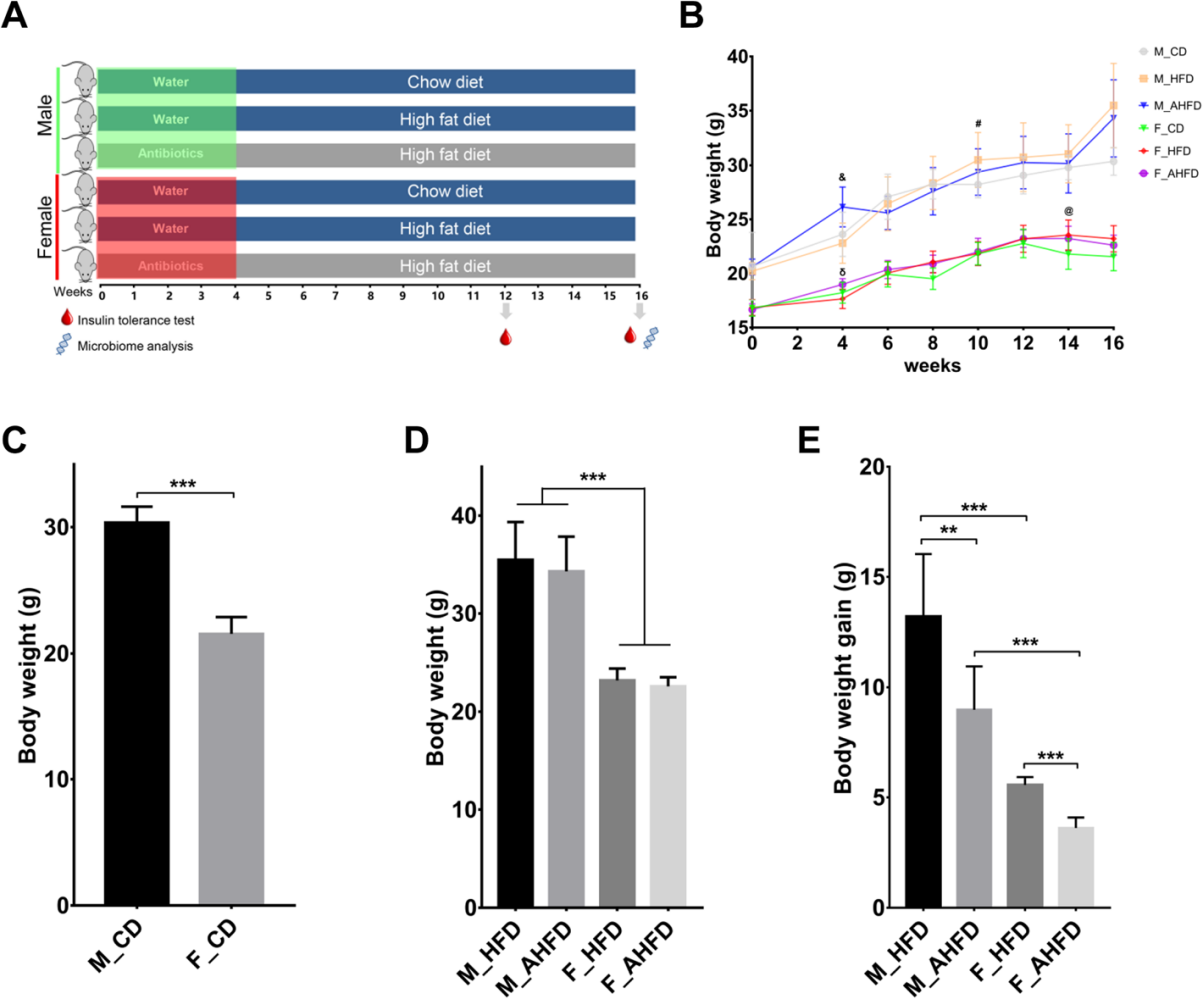
Sex differences in the change in body weight (BW) in response to a high-fat diet (HFD) and antibiotics. **a** Schematic diagram showing the experimental grouping and timeline of mouse model. The male and female mice were divided into three groups, with two groups pretreated with antibiotics for 4 weeks. After 4 weeks of pretreatment, the mice were administered either a chow diet (CD) or HFD for 12 weeks. Then, the insulin tolerance test was conducted at week 12 and week 16. Fecal samples were collected at week 16, which were used for microbial analysis. **b** The BWs of different groups changed over time. The BWs of male (&: *p* < 0.01) and female mice (δ: *p* < 0.05) pretreated with antibiotics for 4 weeks was higher than the control groups. The BW of male mice began to significantly increase at week_10 after HFD (#: *p* < 0.05), while the time for female mice was week_14 (@: *p* < 0.05). The BWs of male and female mice fed either a CD (**c**) or a HFD (**d**) were measured at week 16. The body weight gain (**e**) was also calculated. AHFD group was pretreated with antibiotics for 4 weeks, followed by HFD feeding. **p < 0.01, ***p < 0.001

Four- to 6-week-old male and female C57BL/6 mice (*n* = 60) were purchased from Beijing Vital River Laboratory Animal Technology Co., Ltd. (Beijing, China). The mice were kept in a specific pathogen-free facility at 22 ± 1 °C under a 12-h day and night cycle. Food and water were available ad libitum. The animal protocol used in this study was approved by the Institute of Animal Care and Use Committee (approval nos. SCXK 2012-0001 and 11400700181974). The animal experiments were performed in accordance with the Guidelines for the Care and Use of Laboratory Animals at The First Affiliated Hospital of Nanchang University. After a 1-week acclimation period, male and female mice were randomly divided into two groups: the non-antibiotic-treated and antibiotic cocktail-treated groups [16]. In short, mice in the antibiotic group were given drinking water with 1 g/L ampicillin (Sigma), 1 g/L neomycin sulfate (Sigma), 1 g/L metronidazole (Sigma), and 0.5 g/L vancomycin (Sigma) for 4 weeks to deplete the indigenous gut microbiota. After 4 weeks of pretreatment, the mice were fed either a chow diet (D12450B, Beijing KeAoXieLi Company, Ltd., Beijing, China) or HFD (D12451, Beijing KeAoXieLi Company, Ltd.) (Fig. 1a) (*n* = 10 for each group). The ingredients of these two diets, which are shown in Table 1, were comparable to determine the impact of diet on the gut microbiota [17, 18]. The antibiotic-treated mice were all given a HFD, as the effect of antibiotics on metabolism in mice fed a CD was reported in a previous study [19]. To investigate the effects of an HFD on body weight (BW), body weight gain (BWG) was calculated using the following formula as follows: the weight at week 16 minus the weight at week 4.

**Table 1:** Composition of the experimental diets used in the study

	Chow diet	High-fat diet
Energy(%kcal)		
Protein	20	20
Carbohydrate	70	35
Fat	10	45
Total	100	100
Ingredient (kcal)		
Casein, 80 mesh	800	800
L-cystine	12	12
Corn starch	1260	291
Maltodextrin 10	140	400
Sucrose	1400	691
Soybean oil	225	225
Lard	180	1598
Vitamin mix V1001	40	40
Total	4057	4057

### Glucose homeostasis

After 8 and 12 weeks of feeding different diets, the mice were fasted for 6 h, and the intraperitoneal insulin tolerance test (IPITT) was performed after the mice had been intraperitoneally injected with insulin (0.75 U/kg) [20]. Briefly, the tip of each mouse’s tail was cleaned with alcohol wipes, and then the tail tip distal to the bone was cut 1–2 mm with a surgical scissor. Blood was squeezed from the tail and directly put directly onto a glucose test strip. Then, the blood glucose concentrations were measured using a handheld glucometer (OneTouch Ultra Easy, LifeScan) via tail bleed before (0 min) and after (15, 30, 60, 120 min) the insulin administration. The serum insulin concentration after mice fasted overnight and sacrificed was quantified by ELISA (CrystalChem, Inc.).

### Gut microbiota analysis

Fresh feces were collected before the mice were sacrificed, and total genomic DNA was extracted using the E.Z.N.A. Soil DNA Kit (Omega Bio-Tek, Norcross, GA, USA). The DNA concentration was assessed using a Nanodrop (Thermo Scientific), and the quality was determined by agarose gel electrophoresis. Bacterial 16S rRNA gene sequences spanning the variable regions V3–V4 were amplified using the primer 338F_806R. The amplicons were then extracted from 2% agarose gels, further purified using the AxyPrep DNA Gel Extraction Kit (Axygen Biosciences, Union City, CA, USA) and quantified by QuantiFluor^TM^-ST (Promega, USA). Purified amplicons were pooled in equimolar amounts and subjected to paired-end sequencing (2×300) on an Illumina MiSeq platform according to the standard protocols of Majorbio Bio-Pharm Technology Co. Ltd. (Shanghai, China). The raw sequencing data were deposited into NCBI Sequence Read Archive (SRA, http://www.ncbi.nlm.nih.gov/sra) under accession number SRP218349.

### Bioinformatic analysis of 16S rRNA sequencing data

The raw paired-end sequencing reads obtained from the sequencer were demultiplexed and quality-filtered using Trimmomatic and FLASH. The reads were clustered as operational taxonomic units (OTUs) with the scripts of USEARCH (version 7.0) software with a 97% similarity threshold. Chimeric sequences were identified and deleted. The representative OTU sequences were taxonomically classified against the Silva (SSU128) 16S rRNA database using Ribosomal Database Project (RDP) Classifier (version 2.2) with a confidence threshold of 70%. Within-sample diversity (alpha diversity), as demonstrated by the Shannon index and observed species richness (Sobs), was determined using using Mothur v.1.30.1. Between-sample diversity (beta diversity), which emphasizes differences across samples, was determined by performing nonmetric multidimensional scaling (NMDS) ordination. Using the linear discriminant analysis (LDA) effect size measurements (LEfSe) method, we further identified the bacterial taxa differentially represented between groups.

### Functional annotation

The metagenomes of the gut microbiome were imputed from 16S rRNA sequences with Phylogenetic Investigation of Communities by Reconstruction of Unobserved States (PICRUSt) [21]. This method predicts the gene family abundance from phylogenetic information with an estimated accuracy of 0.8. The closed OTU table was used as the input for metagenome imputation and first rarefied to an even sequencing depth prior to the PICRUSt analysis. Next, the resulting OTU table was normalized by 16S rRNA gene copy number. The gene content was predicted for each individual. Then the predicted functional composition profiles were collapsed into levels 2 and 3 of KEGG database pathways. The output file was further analyzed using the Statistical Analysis of Metagenomic Profiles (STAMP) software package [22].

### Statistical analysis

Data are expressed as the mean ± standard error of the mean (SEM). Differences between two groups with normal distributions were evaluated by Student’s *t* test, and one-way analysis of variance was used to compare differences between more than two groups. The least significant difference (LSD) post hoc test was performed when ANOVA indicated significance. Differences between two groups without normal distributions were evaluated by the Mann-Whitney *U* test, and Kruskal-Wallis *H* test was used to compare differences between more than two groups. The Mann-Whitney *U* test was performed as a post hoc test when the Kruskal-Wallis *H* test indicated significance. The results were analyzed using two-way ANOVA to clarify the effect of each of the factors and their interactions. Statistical analysis was performed using the SPSS 13.0 software, and differences were considered to be statistically significant if *p* < 0.05.

## Results

### Sex differences in BWG in response to a HFD and antibiotic pretreatment

The BW of mice in different groups changed over time (Fig. 1b). The BW of males in the HFD group was significantly increased as early as week 10, while that of females in the HFD group showed an increased BW at week 14, indicating that the males were more susceptible to a HFD than the females. As shown in Fig. 1c, d, the male mice were significantly heavier than the female mice, regardless of whether they were given a CD or HFD. Two-way ANOVA showed the significant main effects of gender [*F*(1,38) = 196.38, *p* < 0.0001] and diet [*F*(1,38) = 20.33, *p* < 0.0001] on BW, while there was no significant effect of antibiotics on BW. There was a significant interaction effect between gender and diet [*F*(1,38) = 5.32, *p* = 0.027]. Moreover, BWG in the male mice was much higher than that in the female mice under HFD feeding conditions (Fig. 1e). Analysis of BWG showed the significant main effects of both gender [*F*(1,39) = 129.09, *p* < 0.0001] and antibiotics [*F*(1,39) = 28.99, *p* < 0.0001]. However, there was no significant interaction effect between these two factors.

Then, we examined the impact of antibiotic pretreatment on HFD-induced alterations in mouse BW. Mouse BW of mice after 4 weeks of antibiotics treatment was significantly increased in both males and females compared to the BW of the control group (Fig. 1b). The BW of mice fed a HFD with antibiotic pretreatment (AHFD) was not significantly different from that of the HFD group regardless of sex (Fig. 1c, d). However, BWG in the AHFD group was significantly lower than that in the HFD group among both males and females, and BWG after HFD feeding was higher in males than in females regardless of antibiotic pretreatment (Fig. 1e).

### Sex differences in metabolic parameters in HFD-fed mice with and without antibiotic pretreatment

**Fig. 2:**
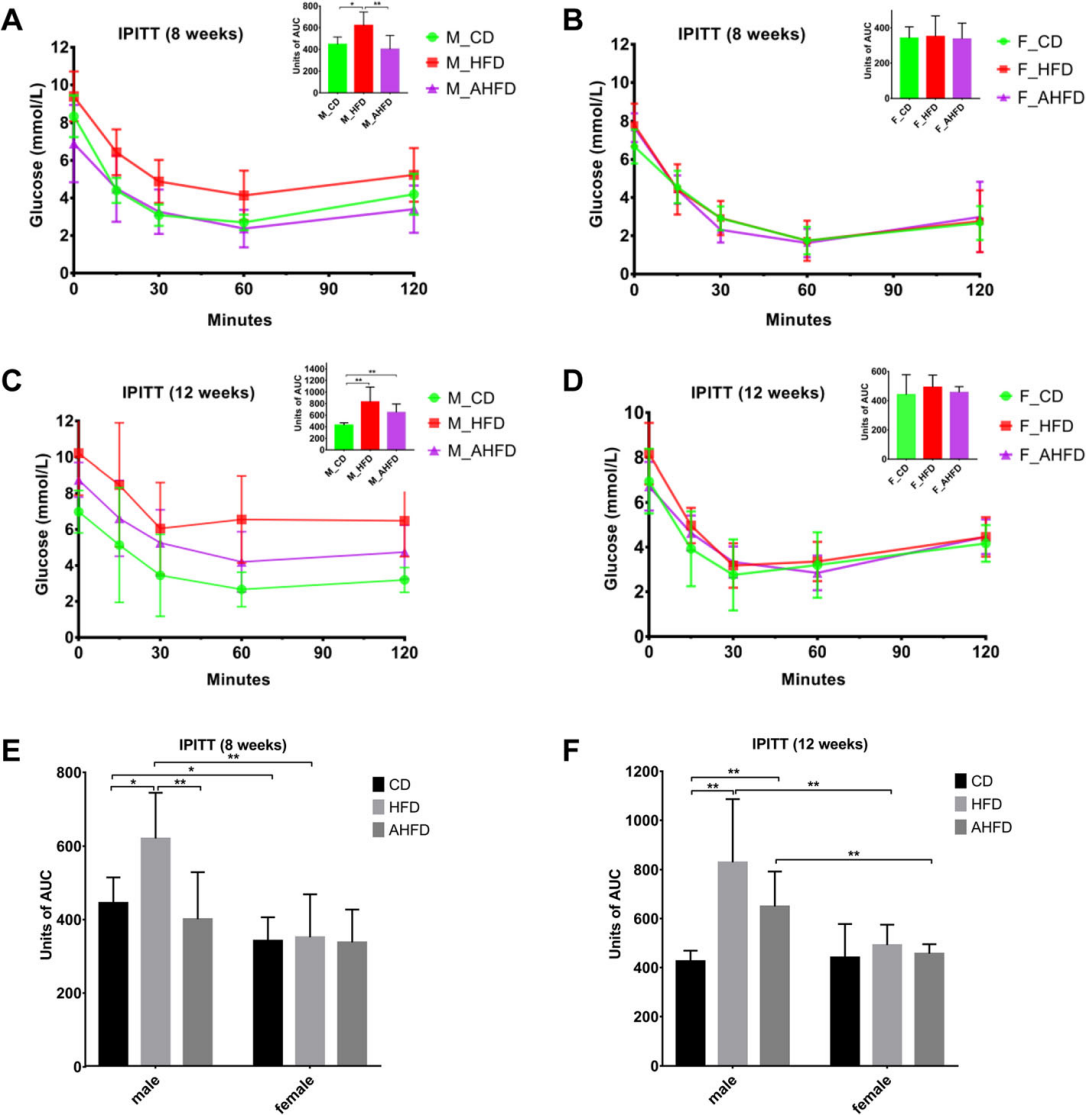
Sex-specific sensitivity to high-fat diet (HFD)-induced insulin resistance with or without antibiotic pretreatment. The intraperitoneal insulin tolerance test was performed in male and female mice after 8 weeks (**a** and **b**) and 12 weeks (**c** and **d**) of HFD feeding. The AHFD group was pretreated with antibiotics for 4 weeks followed by HFD feeding. Statistical analysis of the AUC of male and female mice fed either a chow diet or HFD after 8 weeks (**e**) and 12 weeks (**f**). *p < 0.05, **p < 0.01

We investigated the fasting blood glucose (FBG) level and insulin sensitivity in mice at different time points. Males fed a HFD for 8 weeks displayed more severe insulin resistance compared to those fed a CD, as revealed by the IPITT, while the insulin resistance of the AHFD group was decreased to approximately the extent of the insulin resistance observed in the CD group (Fig. 2a, e). Two-way ANOVA showed the main effects of gender [*F*(1,41) = 14.73, *p* < 0.0001], diet [*F*(1,41) = 6.21, *p* = 0.017], and antibiotics [*F*(1,41) = 10.13, *p* = 0.003] on the IPITT, with significant interaction effects observed between gender and diet [*F*(1,41) = 4.98, *p* = 0.031], as well as gender and antibiotics [*F*(1,41) = 7.84, *p* = 0.008]. However, among male mice, after HFD feeding for 12 weeks, insulin resistance was increased in the AHFD group compared to the CD group and not significantly different compared with that in the HFD group (Fig. 2c, f). Intriguingly, no significant difference in insulin resistance was observed among female mice in the CD, HFD, and AHFD groups at different time points (Fig. 2b, d). Compared to the HFD-fed female group, the HFD-fed male group had significantly higher insulin resistance (Fig. 2e, f).

**Fig. 3:**
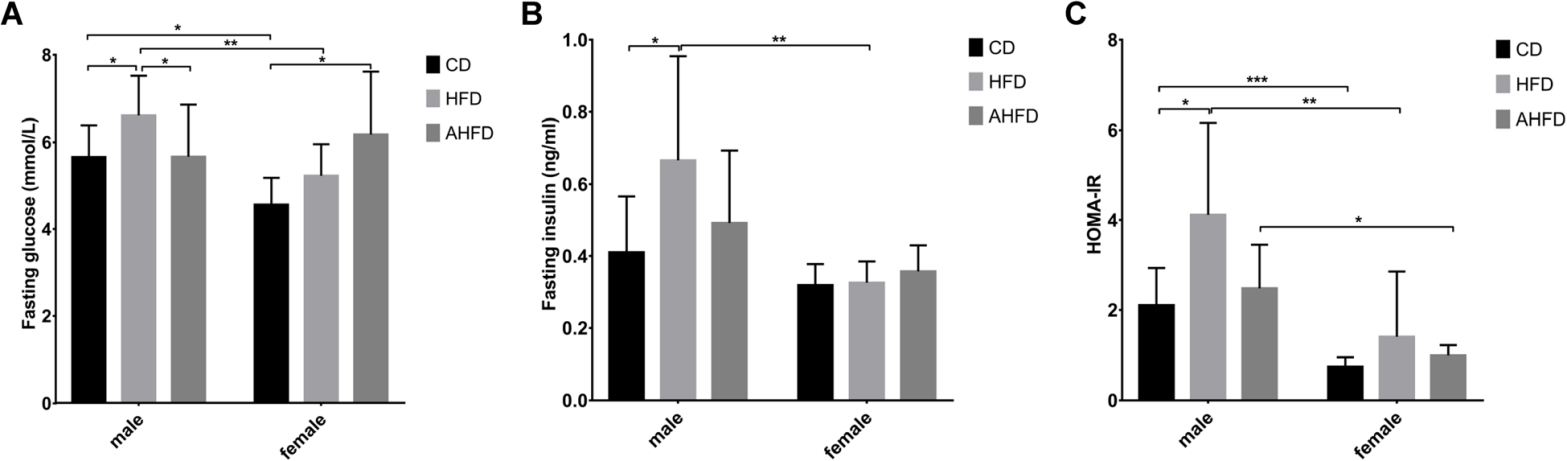
Effect of a high-fat diet (HFD) and antibiotic pretreatment on fasting blood glucose (FBG), insulin, and HOMA-IR levels in different sexes. The FBG (**a**), serum insulin (**b**) and HOMA-IR (**c**) levels were examined in male and female mice with or without antibiotic pretreatment. The AHFD group was pretreated with antibiotics for 4 weeks, followed by HFD feeding. *p < 0.05, **p < 0.01, ***p < 0.001

After 12 weeks of a HFD, the FBG level was significantly elevated in male mice compared to female mice (Fig. 3a). Additionally, we found that among male mice, the FBG level in the AHFD group declined (to a level close to that in the CD group) compared with the FBG level in the HFD group, while the FBG level in HFD-fed female mice was increased with antibiotic pretreatment. Two-way ANOVA showed the main effect of diet [*F*(1,59) = 7.59, *p* = 0.008] but not gender on FBG, and there was no significant interaction effect between gender and diet. Similarly, the serum insulin level was significantly increased in male mice in the HFD group, while there were no differences in serum insulin levels among female mice in the CD, HFD, and AHFD groups (Fig. 3b). We observed the main effects of both gender [*F*(1,43) = 11.37, *p* < 0.0001] and diet [*F*(1,43) = 5.81, *p* = 0.02] on insulin level, and there was also a significant interaction effect between these two factors [*F*(1,43) = 5.21, *p* = 0.027]. The male mice had higher FBG and insulin levels than the female mice when given a HFD (Fig. 3a, b). We calculated the HOMA-IR index of each group, and the results showed that the HOMA-IR index was increased in male mice in the HFD group compared to those in the control group, while no significant difference in HOMA-IR index was observed in female groups. Consistently, the male mice had a higher HOMA-IR index than the female mice independent of diet and antibiotic pretreatment (Fig. 3c). Two-way ANOVA showed the significant main effects of gender [*F*(1,46) = 25.14, *p* < 0.0001], diet [*F*(1,46) = 12.36, *p* = 0.001], and antibiotics [*F*(1,46) = 6.62, *p* = 0.013] on the HOMA-IR index, although there were no significant interaction effects between these factors.

### Sex-specific characterization of the gut microbial composition in mice independent of diet structure

**Fig. 4:**
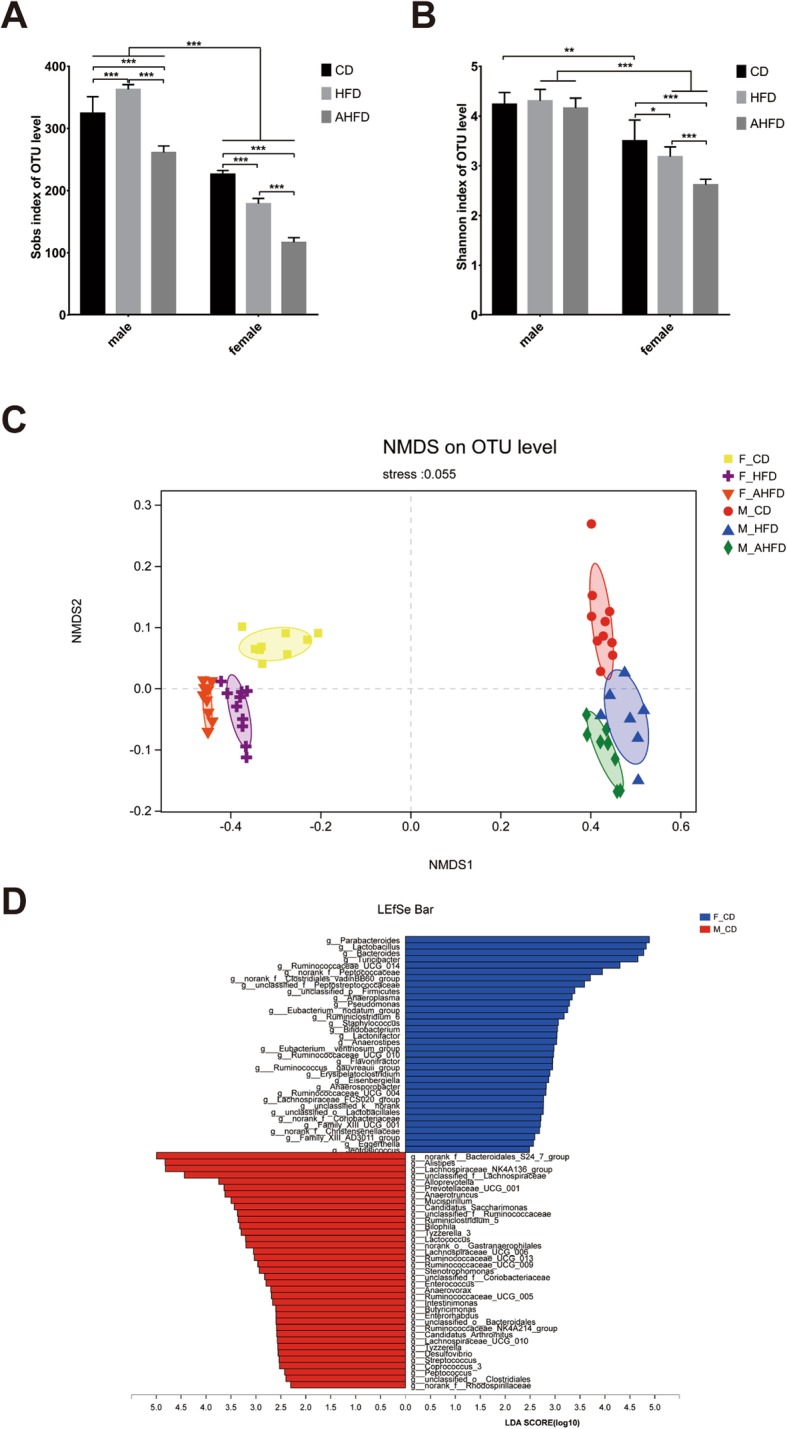
Sex-specific characterization of the gut microbiota using 16S rRNA gene sequencing. Alpha diversity as revealed by the Sobs (**a**) and Shannon (**b**) index was compared between male and female mice fed either a chow diet or high-fat diet. *p < 0.05, **p < 0.01, ***p < 0.001. **c** Bray-Curtis-based nonmetric multidimensional scaling (NMDS) analysis displayed a distinct separation between samples from male and female mice. **d** LEfSe analysis identified the taxa with the most differential abundant between male and female mice. Taxa enriched in females were indicated by a positive LDA score (blue), while taxa enriched in males were indicated by a negative LDA score (red)

16S rRNA gene sequencing of fecal samples revealed that two indexes reflecting species richness and diversity (the Sobs index and Shannon index, respectively) were significantly higher in male mice compared with female mice regardless of CD or HFD feeding (Fig. 4a, b). We observed the significant main effects of both gender [*F*(1,48) = 1202.74, *p* < 0.0001 for Sobs; *F*(1,48) = 264.07, *p* < 0.0001 for Shannon index] and antibiotics [*F*(1,48) = 306.42, *p* < 0.0001 for Sobs; *F*(1,48) = 17.71, *p* < 0.0001 for Shannon index] on Sobs and the Shannon index; however, diet did not have a significant effect. There was a significant interaction effect between gender and diet [*F*(1,48) = 86.34, *p* < 0.0001 for Sobs; *F*(1,48) = 5.49, *p* = 0.023 for Shannon index] as well as gender and antibiotics [*F*(1,48) = 17.62, *p* < 0.0001 for Sobs; *F*(1,48) = 6.17, *p* = 0.017 for Shannon index]. Bray-Curtis-based nonmetric multidimensional scaling (NMDS) analysis at the OTU level showed that the composition of the gut microbiota in male mice was clearly separated from that in female mice regardless of whether they were fed a CD or HFD (Fig. 4c). Interestingly, we also found that the impact of sex on the gut microbiota seems to be greater than that of HFD feeding.

To identify bacterial taxa that were significantly different between female and male mice, a metagenomic biomarker discovery approach (LDA effect size, LEfSe) was used. We found that the beneficial bacteria such as *Parabacteroides*, *Lactobacillus*, *Bacteroides*, and *Bifidobacterium* were significantly enriched in female mice, while sequences from *Bacteroidales_S24-7_group*, *Alistipes*, *Bilophila*, *Desulfovibrio*, *Enterococcus*, *Streptococcus*, and *Peptococcus* were more abundant in male mice (Fig. 4d).

### Sex differences in gut microbial alterations in response to a HFD and antibiotic pretreatment

Gut microbial alterations after 12 weeks of HFD feeding were distinctly different between male and female mice. The Sobs and Shannon index were significantly reduced in female mice fed a HFD compared with those fed a CD , while the Sobs was significantly increased in male mice given a HFD, and no significant difference in the Shannon index was observed between male mice fed a HFD and those fed a CD (Fig. 4a, b). The Sobs was decreased in both male and female mice in the AHFD group compared to the HFD group (Fig. 4a). While the Shannon index was significantly lower in female mice in the AHFD group compared to the HFD group, while there was no significant difference in the Shannon index in male mice between these two groups (Fig. 4b).

**Fig. 5:**
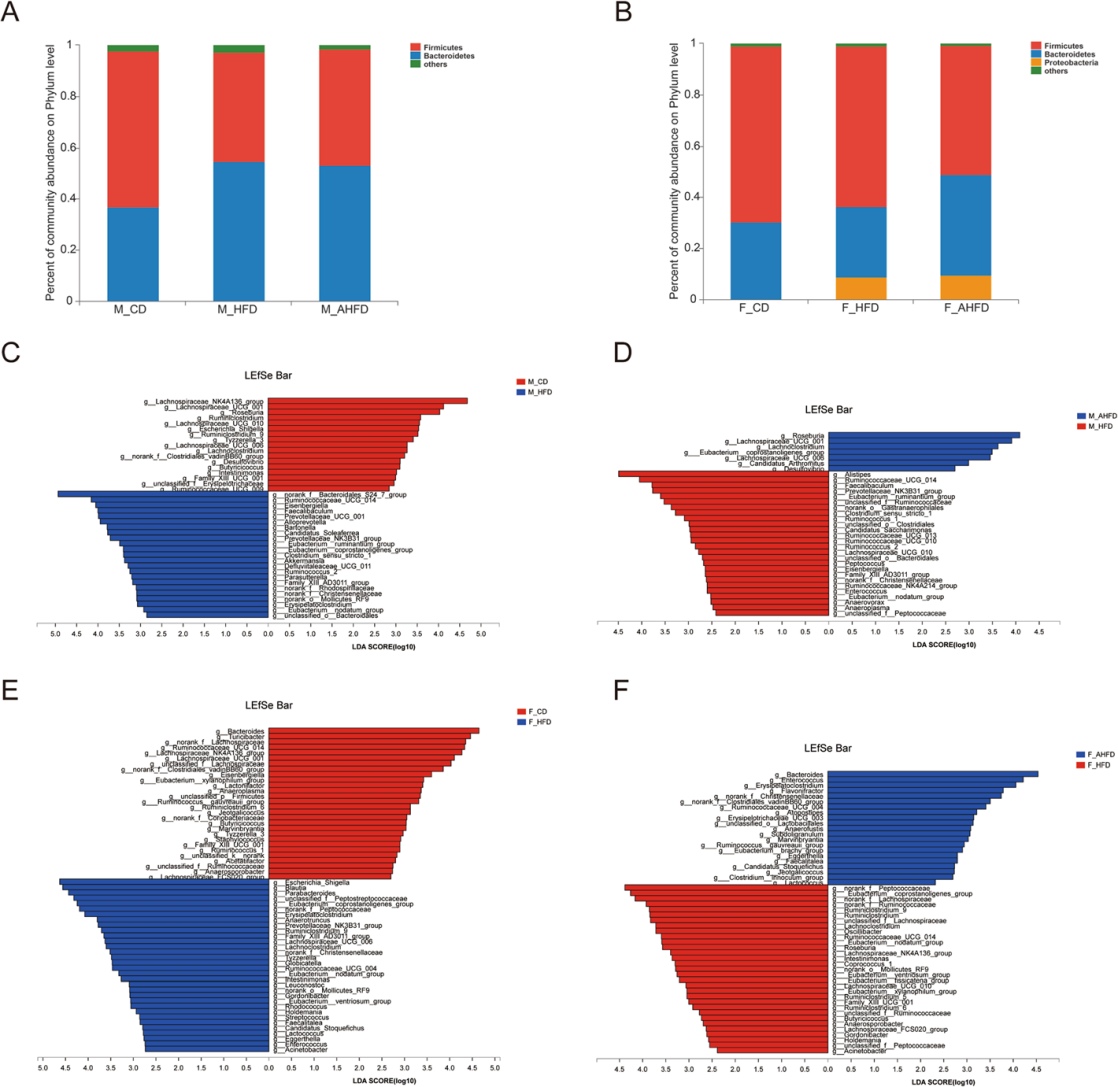
The effect of a high-fat diet (HFD) and antibiotic pretreatment on the composition of the gut microbiota was sex dimorphic. Phylum-level comparison between chow diet (CD) group and HFD group in male (**a**) and female (**b**) mice. LEfSe analysis was used to identify differential genera between CD and HFD group in male (**c**) and female (**e**) mice. Differential genera between HFD group and HFD pretreated with antibiotics (AHFD) group in male (**d**) and female (**f**) were also analyzed

The NMDS analysis showed that the samples from the HFD group clustered separately from those from the CD group, while clear discriminations between both female and male mice in the HFD and AHFD subgroups were observed (Fig. 4c). At the phylum level, HFD-fed male mice had a decreased ratio of *Firmicute*/*Bacteroidetes* while no difference was observed in female mice given HFD (Fig. 5a, b). At the genus level, male mice fed a HFD had a lower abundance of *Lachnospiraceae_NK4A136_group*, *Roseburia*, *Ruminiclostridium*, *Ruminiclostridium_9*, and *Butyricicoccus* than male mice fed a CD, while female mice fed a HFD had a higher abundance of *Escherichia Shigella*, *Blautia*, *Parabacteroides*, and *Eubacterium_coprostanoligenes_group* than female mice fed a CD (Fig. 5c and e). Moreover, the AHFD group of male mice had increased abundance of *Roseburia*, *Lachnoclostridium*, *Eubacterium_coprostanoligenes_group*, and *Lachnospiraceae_UCG_006*, and a decreased the abundance of *Alistipes* than male mice in the HFD group (Fig. 5d). In addition, the relative abundance of *Bacteroides*, *Enterococcus*, and *unclassified_o_Lactobacillales* was enriched in females in the AHFD group, while *norank_f_Peptococcaceae*, *Eubacterium_coprostanoligenes_group*, *Ruminiclostridium_9*, *Lachnoclostridium*, *Roseburia*, and *Butyricicoccus* were more prevalent in females in the HFD group (Fig. 5f).

### Differences in predicted functional pathways of gut microbiota in response to diet and antibiotic pretreatment between male and female mice

**Fig. 6:**
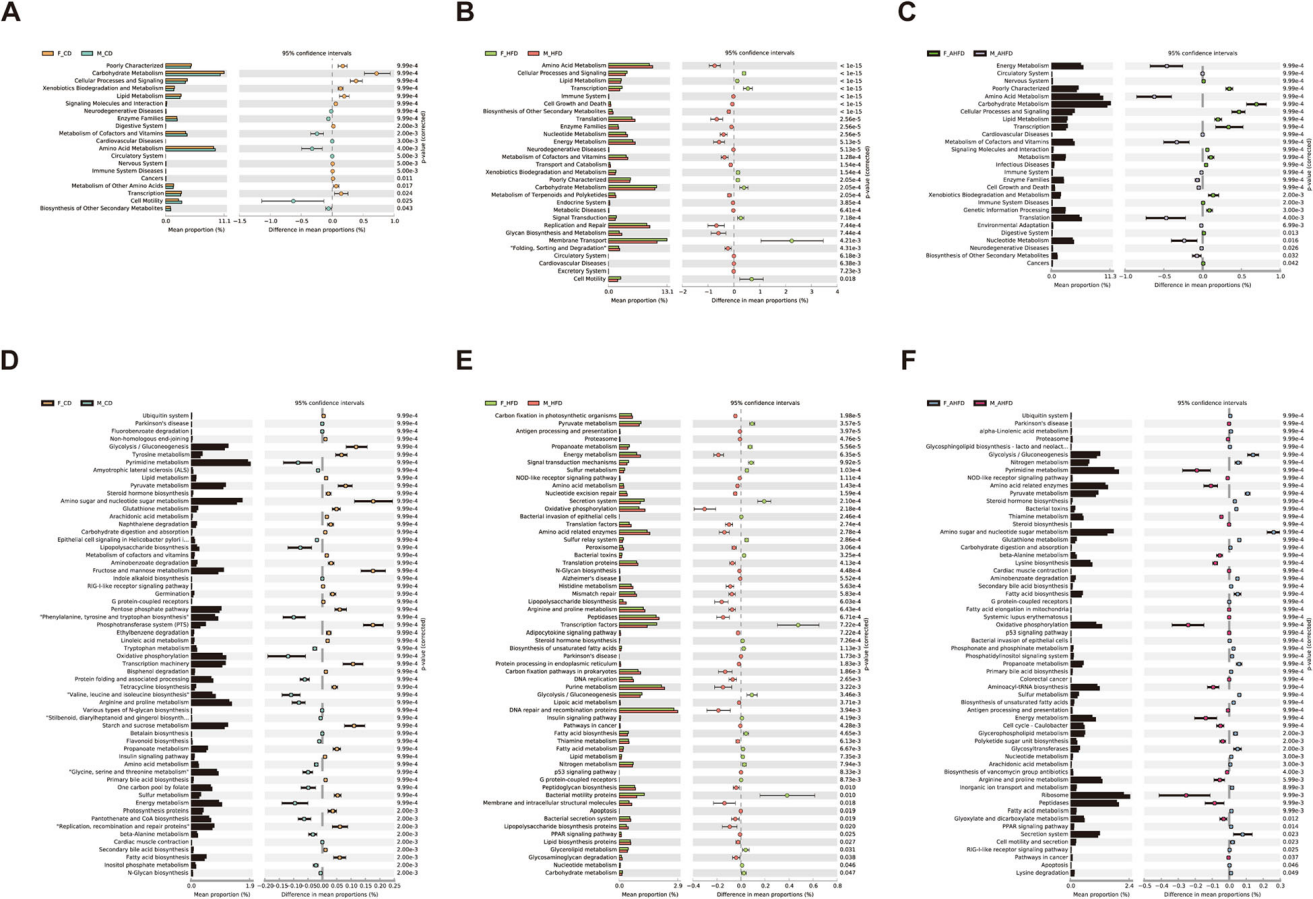
PICRUSt analysis predicted functional composition differences between male and female mice. The predicted metabolic functions of the gut microbiota of male and female mice given either a chow diet (CD) (**a**) or high-fat diet (HFD) (**b**) in level 2 KEGG pathways were generated. Differential enrichment analysis for KEGG level 3 pathways was performed in male and female mice fed a CD (**d**) or HFD (**e**). The sex differences in level 2 (**c**) and level 3 (**f**) KEGG microbial function pathways were also compared between the HFD groups pretreated with antibiotics

Furthermore, we also investigated the functional capacity of the fecal microbiomes of male and female mice. Compared to CD-fed male mice, functional changes in CD-fed female mice included the significantly increased enrichment of predicted KEGG pathways (level 2) involved in carbohydrate metabolism, lipid metabolism, and cellular process and signaling, while the pathways involving cardiovascular diseases and amino acid metabolism were enriched in CD-fed male mice (Fig. 6a). Moreover, we also observed differences in the predicted functional pathways of male and female mice in the HFD group. Compared to HFD-fed male mice, the lipid metabolism, and carbohydrate metabolism pathways were more enriched in HFD-fed female mice, while pathways involving cardiovascular diseases, and metabolic diseases were less enriched (Fig. 6b). Additionally, pathways involving lipid metabolism and carbohydrate metabolism were more enriched in female mice in the AHFD group compared to their male counterparts, while pathways involving cardiovascular diseases and amino acid metabolism were less enriched (Fig. 6c).

Furthermore, we explored the differential predicted functions in level 3 of KEGG pathways. The results showed that KEGG pathways involving lipopolysaccharide biosynthesis were enriched in male mice fed a CD, while the fatty acid biosynthesis and insulin signaling pathway were enriched in female mice fed a CD (Fig. 6d). Under HFD conditions, significantly greater enrichment in the insulin signaling pathway, fatty acid biosynthesis, fatty acid metabolism, lipid metabolism, biosynthesis of unsaturated fatty acids, and carbohydrate metabolism was observed in the female mice compared to male mice (Fig. 6e). Moreover, the pathways including lipopolysaccharide biosynthesis, adipocytokine signaling pathway, lipopolysaccharide biosynthesis proteins, lipid biosynthesis proteins, and peptidoglycan biosynthesis pathways were more enriched in male mice given a HFD than in female mice given a HFD. Similarly, we found that the fatty acid biosynthesis, primary bile acid biosynthesis, secondary bile acid biosynthesis, biosynthesis of unsaturated fatty acids, and fatty acid metabolism pathways were more enriched in AHFD-treated female mice than in AHFD-treated male mice (Fig. 6f).

## Discussion

This study showed sex-specific changes in metabolic parameters in mice when exposed to a HFD and that females exhibited increased resistance to diet-induced metabolic disorders compared to males. 16S rRNA sequencing analysis revealed that the structure of the gut microbiota was influenced by both sex and HFD feeding, with sex having a larger impact than a HFD. These sexually dimorphic differences in the gut microbiota remained significant even after antibiotic pretreatment. Moreover, the responses of males and females to perturbation of the gut microbiota followed by HFD feeding were distinct.

The present study showed that male mice were more susceptible to HFD-induced metabolic disorders than female mice, as revealed by increased body weight gain, and insulin resistance and higher levels of fasting blood glucose. Indeed, sexual dichotomy in metabolic disorders including obesity, metabolic syndrome, and atherosclerosis, is well described; this sexual dichotomy is highly consistent in human and animal models and enables the exploration of the underlying mechanisms [23]. A previous study demonstrated that a HFD and sex were powerful modifiers of metabolic parameters in mice and that the majority of these variables were modified by sex, with males affected by a HFD to a greater degree than females [3].

One of the primary causes of sex differences in metabolic disorders is sex steroid hormones. The female hormone estrogen has been reported to play a fundamental role in the control of energy homeostasis and glucose metabolism, as its deficiency results in metabolic dysfunction predisposing to obesity, metabolic syndrome, and type 2 diabetes [24]. Notably, one of the principal regulators of circulating estrogens is the gut microbiome, which acts through the secretion of β-glucuronidase, an enzyme that deconjugates estrogens into their active forms [25]. Interestingly, we found sex-specific characteristics of the gut microbiota in C57BL/6 mice regardless of diet or antibiotic pretreatment, both of which also affected the composition of the gut microbiota, although to a lesser degree than sex. A recent study in human adults also reported the influence of sex on the biodiversity of the gut microbiota, which remained after adjusting for cardiometabolic parameters [10]. Similar results were observed in animal models. Org et al. showed clear differences in the microbiota composition and diversity between sexes within mice strains and further confirmed that this difference was mediated in part by sex hormones [9]. Moreover, we detected that alterations in the gut microbiota in response to exposure to HFD were different between male and female mice. At the phylum level, the relative abundance of *Firmicutes* decreased, while that of *Bacteroidetes* increased in male mice fed a HFD, while no significant difference was observed in female mice. This finding agrees with a previous study in humans that showed a decreased ratio of *Firmicutes*/*Bacteroidetes* after 6 months of HFD feeding [26]. These diet-microbiota correlations have also been shown to depend on sex in three strains of mice in which specific and similar shifts were observed in both genders [9].

Next, we identified the sex-specific characteristic taxa using LEfSe analysis and observed a higher abundance of *Parabacteroides*, *Lactobacillus*, and *Bifidobacterium* in female mice compared to male mice. Some strains of *Lactobacillus* and *Bifidobacterium* have been recognized as probiotics that have anti-inflammatory effects in vitro and in vivo [27]. For example, oral administration of these bacteria has been found to alleviate HFD-induced obesity and liver steatosis through inhibiting lipopolysaccharide production by the gut microbiota [28]. Consistently, the predictive functional analysis in our study showed that the inflammation-associated pathway lipopolysaccharide biosynthesis was more enriched in male mice than in female mice. Several studies reported that the abundance of *Bacteroides* and *Parabacteroides* was increased in obese mice given metformin or resveratrol, which improved glucose homeostasis probably mediated by modulating the gut microbiota [29, 30]. Our data showed that *Parabacteroides goldsteinii* was the most abundant *Parabacteroides* species in female mice. The latest study by Wu et al. demonstrated that the oral treatment of HFD-fed mice with *Parabacteroides goldsteinii* alleviated obesity and insulin resistance, along with enhanced intestinal integrity and reduced levels of inflammation [31]. Notably, the distinct microbial compositions between male and female mice remained when the mice were exposed to a HFD. Taken together, our results suggest that sex-specific characteristic gut microbiota are associated with the dichotomy sensitivity to HFD-induced metabolic disorders.

To further clarify the role of the gut microbiota in sex-dependent differences in metabolic diseases, both male and female mice were pretreated with antibiotics, and metabolic parameters were then examined after HFD feeding. Intriguingly, the male and female mice displayed different responses to a HFD after the gut microbiota was decreased with 4 weeks of antibiotic treatment. As revealed by the IPITT, insulin resistance was partially relieved in male mice pretreated with antibiotics, while no significant difference in insulin resistance was observed in female mice. Moreover, we found that the FBG levels in male and female mice given antibiotics followed by a HFD changed in opposite ways, with FBG levels elevated in female mice and deceased in male mice. 16S rRNA sequencing analysis showed the depletion of *Roseburia*, *Ruminiclostridium*, and *Lachnoclostridium*, which have been reported to produce short-chain fatty acids (SCFAs), in HFD-fed female mice with antibiotic pretreatment [32–34]. SCFAs, formed by microbial fermentation, are believed to play a beneficial role in host metabolism including body weight control and the improvement of insulin sensitivity [35]. In addition, we observed that the abundance of *Roseburia* was decreased in male mice in the HFD group but increased in those that pretreated with antibiotics. In contrast, the abundance of *Roseburia* was reduced in female mice given antibiotic pretreatment followed by HFD feeding. Our data showed that the changes in *Roseburia* were consistent with beneficial glucose metabolism. This agrees with previous studies that presented *Roseburia* as probiotic bacteria that maintained intestinal physiology and immune homeostasis through producing SCFAs [36]. A recent study by Gao et al. also uncovered sex-dependent alterations in the gut microbiota and metabolites in mice in response to different antibiotics [37]. Collectively, these results show that antibiotic pretreatment eliminates some beneficial microbes that play a role in the resistance against HFD-induced metabolic disorders in females, while some pathogenic microbes that promote these diseases are eliminated after antibiotic treatment in males.

### Perspectives and significance

In summary, our study shows that the composition of the gut microbiota was distinct between male and female mice, with an increased abundance of *Parabacteroides*, *Lactobacillus*, and *Bifidobacterium* observed in females compared to males. Environmental factors (a HFD and antibiotics) also affected the structure of the gut microbiota, although to a lesser degree than sex. Sex-specific characteristics of the gut microbiota may be associated with the different sensitivities of male and female mice to metabolic disorders in response to a HFD. Further studies are warranted to elucidate the causal role of the gut microbiota in sex dichotomy in metabolic diseases. Overall, these findings improve our understanding of sex differences in the gut microbiome and its alterations after HFD feeding or antibiotic treatment. These findings also reveal the opposite manifestations of a HFD in male and female mice with short-term destruction of the gut microbiota, indicating the sex-dependent effects of antibiotics on metabolic disorders.

## Abbreviations

AHFD: Antibiotics treatment followed by high-fat diet
BW: Body weight
BWG: Body weight gain
CD: Chow diet
FBG: Fasting blood glucose
HFD: High-fat diet
HOMA-IR: Homeostasis model assessment-insulin resistance
IPITT: Intraperitoneal insulin tolerance test
KEGG: Kyoto Encyclopedia of Genes and Genomes
LEfSe: Linear discrimination analysis coupled with effect size
NMDS: Nonmetric multidimensional scaling
OTU: Operational taxonomic unit
PICRUSt: Phylogenetic Investigation of Communities by Reconstruction of Unobserved States
rRNA: Ribosomal RNA
SCFAs: Short-chain fatty acids
